# Do socioeconomic differences in tobacco use exist also in developing countries? A study of Ghanaian adolescents

**DOI:** 10.1186/1471-2458-10-758

**Published:** 2010-12-08

**Authors:** David Doku, Leena Koivusilta, Susanna Raisamo, Arja Rimpelä

**Affiliations:** 1 Tampere School of Public Health, University of Tampere, FI-33014 University of Tampere, Tampere, Finland; 2 Department of Social Research, Faculty of Social Sciences, University of Turku, FI-20014 University of Turku, Turku, Finland

## Abstract

**Background:**

In Western countries, tobacco use is most prevalent among adolescents in lower socioeconomic groups. The association between socioeconomic status (SES) and tobacco use among adolescents in developing countries is unexplored. Using multiple SES measures, we investigated this association among adolescents in Ghana.

**Method:**

A school-based survey of a representative sample of 13-18-year-old Ghanaians (N = 1,165, response rate = 89.7%) was conducted in three regions, in 2008. Logistic regression analysis was used to evaluate the relationship of smoking, tawa (smokeless tobacco) use with familial SES (parental occupation and education, material affluence scale, family structure), an adolescent's individual social position (school performance, plans after graduation) and inter-generational social mobility (predicted by the differences of familial and individual positions).

**Results:**

Socioeconomic differences existed in tobacco use whether measured by familial SES or individual social position with higher prevalence in lower socioeconomic groups. Low father's education and living in a non-nuclear family were associated with both forms of tobacco use while low material affluence was associated with tawa use only; individual social position measured by plans after graduation was the strongest predictor of both smoking and tawa use. Inter-generational downward social mobility and particularly staying in low SES was related to both forms of tobacco use.

**Conclusions:**

Similar to Western countries, lower SES is related to an adolescent's tobacco use also in developing countries. Cumulative socioeconomic disadvantage over generations increases the probability of tobacco use.

## Background

Socioeconomic inequality and its impact on health is a growing global public health concern [1]. Smoking has been identified as the single biggest cause of inequality in morbidity and mortality between rich and poor people in many countries [2]. Studies from Western countries have reported an association between socioeconomic status (SES) and smoking to the disadvantage of those in lower SES groups [3]. Studies among adolescents have shown the same pattern, with some exceptions where the association was found only for some ages, genders or SES indicators [4-9]. In developing countries among adolescents, the relationship between socioeconomic factors and smoking is unknown. In this study, we explore this relationship among adolescents in Ghana, a developing country in sub-Saharan Africa.

Unlike in Western and many other developing countries, the prevalence of smoking in sub-Saharan Africa is relatively low both among adolescents as well as adults, based on the scanty information available [10]. In Ghana, a small study of urban adolescents shows that lifetime cigarette use was 7.5% [11] and among adults, in one region, 4% were current smokers [12]. Thus the prevalence of smoking is relatively low despite a long history of tobacco cultivation and manufacturing [13], but still Addo et al. [14] found that the current prevalence of tobacco use among civil servants in the capital city of Accra represents a rise over a thirty year period. On the other hand, traditionally, the population has used smokeless tobacco, tawa, but how common this is at the population level or how it relates to SES is not known.

In Ghana, reminiscent of most African countries, there are very little or no tobacco control measures and accessibility as well as availability to minors are unrestricted [11], except on religious or moral grounds. Therefore in view of the little or no tobacco control measures, we expect lower tobacco use among adolescents in the higher socioeconomic groups, who are likely to be favoured by any available health education, parental education and other socio-cultural factors, but higher among those in the lower socioeconomic groups, resulting in socioeconomic differences in tobacco use similar to those found in Western countries.

Assessments of an adolescent's SES should take into account the transitional nature of adolescence and should be conceptualized in two dimensions: familial SES, reflecting the social class of origin, and the adolescent's individual social position in relation to his/her peers [5-9]. The individual social position measured by school career or school performance, predicts education in adulthood [15]. In addition to SES, inter-generational social mobility has been shown to relate to health behaviours including smoking [16-18]. Inter-generational social mobility can be conceptualized as the transition between familial (original) SES in childhood and individual (achieved) social position in adulthood.

The aim of this study was to investigate socioeconomic differences in smoking and tawa use among Ghanaian adolescents using multiple SES measures which assess familial SES and the adolescent's individual social position. Based on these two dimensions, we also explore how the inter-generational social mobility relates to tobacco use.

## Methods

### Data

A cross-sectional survey was conducted from June to August 2008 on health behaviours and lifestyles of school-aged adolescents in three administrative regions in Ghana. Thirty schools were randomly sampled, ten per region, from Eastern (total number of schools in the region = 2924), Greater Accra (total number of schools in the region = 1825) and Volta Regions (total number of schools in the region = 2184). The Ghana Education Service's School Health Programme register of schools in the country was the source of the sampling frame. The sampling was done as follows: First, ten schools were randomly selected so that they comprised of four public Junior High Schools (total number in the three regions = 5325), two private Junior High Schools (total number in the three regions = 1395), three public Senior High Schools (total number in the three regions = 171) and one private Senior High School (total number in the three regions = 47) in each region in order to reflect the school types in Ghana. Second, in each school, all students whose names were found in the class attendance register of the randomly selected classes were eligible to participate in the survey. The eight page questionnaire was anonymous and self-administered. It was designed to exclude any information that will reveal the identities of the participants. One trained supervisor was assigned to each classroom during the questionnaire administration to address pupils' concerns when necessary. The survey commenced simultaneously in all the participating classes in a given school. Participants were asked to drop their questionnaires in an envelope placed in front of the class on completion. The study protocol was approved by the ethical committee of the Ghana Health Service Research Unit in Accra, Ghana.

The characteristics of the respondents are presented in Table 1. Out of the 1566 respondents who completed the questionnaire, only 13-18-year-old students were included in this study (N = 1165). They comprised of 41.5% (483) boys and 55.3% (644) girls. The mean ages for boys and girls were 15.8 years and 15.9 years, respectively. The response rate was 89.7% (the sample was based on academic year's register of pupils). Only one pupil denied answering. A convenient sample of 127 non-students in the same age group showed similar pattern of responses for most of the key indicators we measured in our survey. Among this group 3.6% and 7.3% were tawa users and smokers, respectively.

**Table 1:** Distributions (%) of the characteristics of the study subjects by gender

Variable	Boys (N = 483)	Girls (N = 644)	P-value for gender difference
**Age (yr)**			0.370
13	9.5	6.7	
14	14.1	13.4	
15	17.2	20.7	
16	19.7	21.6	
17	21.1	21.4	
18	18.4	16.3	
**Material affluence scale (N = 1097)**			0.001
Poorest	21.9	16.6	
Second poorest	23.4	14.9	
Average	20.7	19.4	
Affluent	13.7	21.3	
Most affluent	15.9	21.3	
Missing	5.0	6.5	
**Family structure (N = 1159)**			0.010
Both parents dead	5.2	2.0	
Only one parent alive	18.2	16.7	
Both parents alive but not living together	22.2	21.2	
Nuclear family	54.0	59.5	
Missing	0.4	0.6	
**Father's education (N = 1103)**			0.008
Illiterate	9.7	6.5	
Primary education	34.6	29.5	
Secondary education	28.2	33.5	
Tertiary	22.4	24.8	
Missing	5.2	5.6	
**Mother's education (N = 1116)**			0.001
Illiterate	18.2	14.6	
Primary education	46.6	40.1	
Secondary education	22.8	30.4	
Tertiary	7.5	11.2	
Missing	5.0	3.7	
**Father's occupation (N = 1022)**			0.060
Low grade	65.4	56.7	
High grade	25.3	29.3	
Missing	9.3	14.0	
**Mother's occupation (N = 1092)**			0.017
Low grade	86.3	80.3	
High grade	8.1	12.9	
Missing	5.6	6.8	
**School performance (N = 1158)**			<0.001
Low	8.5	16.8	
Middle	48.4	54.7	
High	42.2	28.1	
Missing	0.8	0.5	
**Plans after graduation (N = 1157)**			0.028
Won't continue schooling	14.5	10.1	
Continue schooling	84.3	89.9	
Missing	1.2	0.3	
Mobility 1* **(N = 1090)**			<0.001
Stable (low)	9.9	5.7	
Downward	3.5	3.7	
Upward	34.4	25.5	
Stable (high)	46.2	58.3	
Missing	6.0	6.8	
Mobility 2** **(N = 1095)**			0.005
Stable (low)	7.5	5.4	
Downward	6.0	3.9	
Upward	36.2	30.6	
Stable (high)	43.9	54.2	
Missing	6.4	5.9	
**Tawa use**			0.018
Yes	6.8	3.7	
No	87.4	91.6	
Missing	5.8	4.7	
**Smoking**			0.122
Yes	7.5	4.3	
No	85.3	88.5	
Missing	7.2	7.1	
**Paternal smoking**			0.195
Ever/current	5.4	7.6	
Never	82.4	84.2	
Missing	12.2	8.2	
**Maternal smoking**			0.027
Ever/current	3.7	1.7	
Never	88.2	93.2	
Missing	8.1	5.1	

*Mobility from assigned socioeconomic status measured by material affluence scale to achieved social position measured by plans after graduation.

** Mobility from assigned socioeconomic status measured by father's education to achieved social position measured by plans after graduation.

### Indicators of socioeconomic status

#### Indicators of familial socioeconomic status

A material affluence scale (MAS) of five categories (poorest, poor, average, affluent and most affluent) was used based on our previous research [19]. The items on which the scale was based covered three aspects of material circumstances: household assets (e.g. television) and housing characteristics (e.g. types of house), other assets (e.g. farm ownership) and school related indicators (e.g. working, other than doing household chores, in the morning before going to school). Material affluence mirrors the lack or availability of the resources and goods necessary for decent living in relation to what is generally available in the society [20]. Various kinds of scales measuring material affluence have been constructed to capture the amount of these kinds of resources available in the families [19,21]. The items of the scales are meant to envelop the key aspects of wealth as well as the material circumstances of the family.

Family structure was measured in four categories (nuclear family, both parents alive but not living together, only one parent alive, or both parents dead). Adolescents living in a family other than where both parents were alive and living together were regarded as socially disadvantaged.

Father's, mother's or other guardian's highest level of education were categorised into illiterate, basic education, secondary education and tertiary education according to the classification of the Ghanaian educational system. Parental occupational status was measured by respondents reporting their father's, mother's, or other guardian's occupation or employment status. These were categorised in grades A (chief in rank), B (professional and managerial), C (professional non managerial), D (skilled manual), E (unskilled manual) and unemployed according to grades in the Ghana Civil Service (Head of Civil Service 2000) None of the respondents fell into the A category. We stratified grades B and C as high grade and grades D, E and unemployed as low grade in the analysis.

#### The adolescent's individual social position

Adolescents indicated their school performance in the previous term examination. These were coded into three categories high (excellent, very good), middle (good), and low (average, poor). Adolescents indicated their plans after graduation from the current level of schooling (continue schooling, learn a trade, look for job and not sure). These were coded as continue schooling and not continue schooling (learn a trade, look for job or not sure).

#### Predicted inter-generational social mobility

Two measures of inter-generational social mobility (upward mobility, stable high, stable low and downward mobility) were used. Two combinations of social class of origin (measured by MAS and father's education) and achieved social position (measured by plans after graduation) were computed. Mobility 1: MAS was categorised into High (3 = top 20%), Medium (2 = next 40%) and Low (1 = lowest 40%) while plans after graduation was categorised as continue schooling (1) and not continue schooling (0). Adolescents were classified as socially stable in the low SES (stable in low SES), if MAS = 1 and Plans after graduation = 0. And if MAS = 2 and Plans after graduation = 1 or if MAS = 3 and Plans after graduation = 1, they were classified as socially stable in the high SES (stable in high SES). Adolescents were classified as upwardly mobile, if MAS = 1 and Plans after graduation = 1. Adolescents were classified as downwardly mobile, if MAS = 2 and Plans after graduation = 0 or if MAS = 3 and Plans after graduation = 0, Table 1.

Mobility 2: Father's education was categorised into High (3 = tertiary education), Middle (2 = secondary education) and Low (1 = illiterate or primary education). Adolescents were classified as socially stable in the low SES (stable in low SES), if father's education = 1 and Plans after graduation = 0. And if father's education = 2 and Plans after graduation = 1 or if father's education = 3 and Plans after graduation = 1, they were classified as socially stable in the high SES (stable in high SES). Adolescents were classified as upwardly mobile, if father's education = 1 and Plans after graduation = 1. Adolescents were classified as downwardly mobile, if father's education = 2 and Plans after graduation = 0 or if father's education = 3 and Plans after graduation = 0, Table 1.

#### Indicators of tobacco use

Smokers were adolescents who had ever smoked a cigarette. Tawa users were those who had ever tried tawa. Tawa comes in two forms: *Fine-grain tawa*-tobacco that often comes in teabag-like pouches that users "pinch" or "dip" between their lower lip and gum, allow it sit there and spit out the juice and *chewing tawa***- **tobacco which comes in shredded or twisted tobacco leaves that users put between their cheek and gum, chew it and spit out the juice.

Parental smoking was based on adolescents' responses to two separate questions regarding their mothers' and fathers' smoking measured in five categories (father or mother smoked at present, had never smoked, had smoked but had stopped, couldn't say anything about parental smoking or had no father or mother). Dichotomous (never vs ever/current smokers) variables were made for maternal and paternal smoking.

The proportions of missing data were relatively low for all the indicators (Table 1). The proportion of tawa users and smokers were 5.7% and 6.6%, respectively.

### Statistical analysis

Pearson's Chi-square tests were used to test the associations between gender and each of the studied variables. Logistic regression analysis was used to model the associations between the socioeconomic indicators and tobacco use. The strength of the associations was expressed by odds ratios (OR) and 95% confidence intervals (CI). First, bivariate models (Model1) were fitted including each of the socioeconomic measures one at a time, controlling for age and gender. Second, multivariate logistic regression models were used to test whether individual SES measures were independently predictive of tobacco use. Model 2 included age, gender and all the statistically significant socioeconomic indicators and then Model 3 comprising of the indicators in Model 2 plus parental smoking. For the social mobility analyses models 2 was adjusted for family structure and model 3 was adjusted for family structure and parental smoking. In all analyses, those with the highest socioeconomic advantages were used as the reference categories.

## Results

### Tobacco use by familial socioeconomic status

A lower level of material affluence was associated with the likelihood of tawa use but the association was not statistically significant with smoking. Adolescents who lived in family types other than the nuclear family were more likely to smoke or use tawa compared to those who lived in the nuclear family. Lower paternal education predicted both smoking and tawa use (Table 2, Model 1). Adolescents whose fathers had primary education were more likely to use tawa compared to those whose fathers had tertiary education, albeit at borderline statistical significance. Adolescents who had illiterate fathers were more likely to smoke than those whose fathers had tertiary education. There were no statistically significant associations between tobacco use and mother's education, and father's or mother's occupation. In multivariate analysis, material affluence independently predicted tawa use when the effects of the other statistically significant familial socioeconomic measures (Table 2, Model 2) and parental smoking were controlled for (Table 2, Model 3). Similarly, family structure independently predicted smoking and tawa use.

**Table 2:** Odds ratios (OR) and their 95% confidence intervals (CI) of tobacco use by parental socioeconomic measures among adolescents, statistically significant odds ratios in bold.

Socioeconomic indicator	Tawa use			Smoking		
	Model 1	Model 2	Model 3	Model 1	Model 2	Model 3
	OR (95% CI)	OR (95% CI)	OR (95% CI)	OR (95% CI)	OR (95% CI)	OR (95% CI)
**MAS**					*	*
Most affluent	1.0	1.0	1.0	1.0		
Affluent	0.4 (0.1-2.2) P = 0.303	0.4 (0.1-2.1) P = 0.280	0.4 (0.1-2.1) P = 0.291	0.3 (0.1-3.6) P = 0.093		
Average	1.6 (0.5-4.9) p = 0.432	1.3 (0.4-4.2) P = 0.684	1.4 (0.4-4.6) P = 0.0.567	1.4 (0.5-3.6) P = 0.439		
Second poorest	**4.6 (1.7-12.5)** P = 0.003	**4.0 (1.4-11.3)** P = 0.009	**3.1 (1.0-9.1)** P = 0.040	2.2 (0.9-5.3) P = 0.066		
Poorest	**3.5 (1.2-9.9)** P = 0.016	**3.1 (1.0-9.4)** P = 0.041	**3.2 (1.0-9.5)** P = 0.042	1.6 (0.6-3.9) P = 0.333		
**Family structure**						
Nuclear family	1.0	1.0	1.0	1.0	1.0	1.0
Both parents alive but not together	1.4 (0.7-3.0) P = 0.362	1.6 (0.7-3.7) P = 0.205	1.6 (0.7-3.8) P = 0.213	1.4 (0.7-2.8) P = 0.325	1.3 (0.6-2.7) P = 0.446	1.3 (0.6-2.8) P = 0.420
Only one parent alive	**2.4 (1.2-4.9)** P = 0.011	**2.7 (1.3-5.8)** P = 0.010	2.2 (1.0-5.0) P = 0.054	**2.5 (1.3-4.7)** P = 0.005	**2.2 (1.1-4.3)** P = 0.021	**2.5 (1.3-4.9)** P = 0.006
Both parents dead	**6.9 (2.8-17.2)** P < 0.001	**7.7 (2.7-21.4)** P < 0.001	**6.1 (2.0-18.3)** P < 0.001	**6.1 (2.5-15.0)** P < 0.001	**4.1 (1.5-11.4)** P = 0.006	**5.6 (2.1-14.8)** P < 0.001
**Father's education**			*			*
Tertiary education	1.0	1.0		1.0	1.0	
Secondary education	1.4 (0.6-3.3) P = 0.190	0.6 (0.1-2.1) P = 0.463		1.4 (0.6-2.9) P = 0.411	1.4 (0.6-2.9) P = 0.418	
Primary education	**2.3 (1.0-5.0)** P = 0.041	1.0 (0.4-2.5) P = 0.913		1.0 (0.5-2.3) P = 0.889	0.9 (0.4-2.1) P = 0.889	
Illiterate	2.0 (0.7-5.9) P = 0.425	1.2 (0.5-2.9) P = 0.694		**3.0 (1.3-7.3)** P = 0.013	2.4 (1.0-5.9) P = 0.060	
**Mother's education**		*	*		*	*
Tertiary education	1.0			1.0		
Secondary education	0.7 (0.2-1.9) P = 0.437			0.4 (0.1-1.3) P = 0.138		
Primary education	1.0 (0.4-2.4) P = 0.936			1.1 (0.4-2.9) P = 0.774		
Illiterate	1.3 (0.5-3.7) P = 0.591			0.8 (0.3-2.5) P = 0.783		
**Father's occupation**		*	*		*	*
High grade	1.0			1.0		
Low grade	1.3 (0.7-2.5) P = 0.367			1.2 (0.7-2.2) P = 0.526		
**Mother's occupation**		*	*		*	*
High grade	1.0			1.0		
Low grade	1.1 (0.4-2.6) P = 0.873			0.6 (0.3-1.4) P = 0.647		

Model 1 = socioeconomic measure + age + gender (bivariate).

Model 2 = age + gender + statistically significant socioeconomic measures in model 1.

Model 3 = age + gender + statistically significant socioeconomic measures in model 2 + paternal smoking +maternal smoking.

* Not included in the model.

#### Tobacco use by individual social position

There were striking differences in tobacco use by plans after graduation but not by school performance (Table 3). Adolescents who did not have any plans of continuing schooling after graduating were more likely to smoke or use tawa than those who planned to further their education. In multivariate analysis, plans after graduation independently predicted both smoking and tawa use even after controlling for MAS, family structure and father's education (Table 3, Model 2) and parental smoking (Table 3, Model 3).

**Table 3:** Odds ratios (OR) and their 95% confidence intervals (CI) of tobacco use by measures of adolescents' individual social position, statistically significant odds ratios in bold.

Indicator of individual social position	Tawa use			Smoking		
	Model 1	Model 2	Model 3	Model 1	Model 2	Model 3
	OR (95% CI)	OR (95% CI)	OR (95% CI)	OR (95% CI)	OR (95% CI)	OR (95% CI)
School performance		*	*		*	*
Above average	1.0			1.0		
Average	0.4 (0.1-1.3) P = 0.149			0.6 (0.2-1.4) P = 0.244		
Below average	0.6 (0.4-1.2) P = 0.151			0.7 (0.4-1.2) P = 0.202		
Plans after graduation						
Continue school	1.0	1.0	1.0	1.0	1.0	1.0
Not continue school	**3.7 (2.0-6.7)** P < 0.001	**3.2 (1.7-6.3)** P < 0.001	**2.7 (1.3-5.6)** P < 0.001	**4.0 (2.5-7.0)** P < 0.001	**3.6 (1.6-6.8)** P < 0.001	**3.4 (1.6-6.8)** P < 0.001

Model 1 = socioeconomic measure + age + gender (bivariate).

Model 2 = age + gender + material affluence scale + family structure + father's education.

Model 3 = age + gender + material affluence scale + family structure + father's education + paternal smoking +maternal smoking.

* Not included in the analysis.

### Tobacco use and adolescents' predicted inter-generational social mobility

Tawa use and smoking were related to both downward social mobility and stable low SES whether mobility was measured by material affluence scale or father's education compared to being stable in the high SES (Table 4). In a multivariate analysis, tawa use and smoking were independently related to downward social mobility and particularly being stable in low SES by both indicators of social mobility, after adjusting for family structure (Table 4, Models 2) and parental smoking (Table 4, Models 3). The only exception was that relationship between tawa use and downward mobility disappeared after controlling for parental smoking. Upwardly mobile adolescents did not differ from those stable in the high SES by smoking or tawa use.

**Table 4:** Odds ratios (OR) and their 95% confidence intervals (CI) of tobacco use by adolescents' predicted social mobility, statistically significant odds ratios in bold.

Social mobility indicator	Tawa use			Smoking		
	Model 1	Model 2	Model 3	Model 1	Model 2	Model 3
	OR (95% CI)	OR (95% CI)	OR (95% CI)	OR (95% CI)	OR (95% CI)	OR (95% CI)
Mobility 1* (N = 1090)						
Stable in low SES	**6.5 (2.7-15.7)** P < 0.001	**6.0 (2.4-14.8)** P < 0.001	**6.1 (2.4-15.3)** P < 0.001	**5.2 (2.2-12.1)** P < 0.001	**4.8 (2.0-11.5)** P < 0.001	**4.7 (1.8-12.1)** P = 0.001
Downwardly mobile	**2.9 (1.4-6.2)** P = 0.006	**2.4 (1.1-5.3)** P = 0.025	2.1 (0.9-4.8) P = 0.064	**3.1 (1.6-6.2)** P = 0.001	**2.5 (1.2-5.0)** P = 0.009	**2.6 (1.2-5.4)** P = 0.011
Upwardly mobile	1.3 (0.6-2.9) P = 0.492	1.2 (0.5-2.8) P = 0.596	1.1 (0.5-2.7) P = 0.742	0.6 (0.2-1.2) P = 0.312	0.5 (0.2-1.4) P = 0.229	0.6 (0.2-1.7) P = 0.391
Stable in high SES	1.0	1.0	1.0	1.0	1.0	1.0
Mobility 2** (N = 1095)						
Stable in low SES	**6.2 (2.8-13.7)** P < 0.001	**5.0 (2.2-11.5)** P < 0.001	**4.6 (2.0-10.9)** P < 0.001	**4.2 (1.9-9.1)** P < 0.001	**3.5 (1.6-7.8)** P = 0.002	**3.4 (1.4-8.2)** P = 0.006
Downwardly mobile	**3.3 (1.2-8.8)** P = 0.017	2.8 (1.0-7.5) P = 0.046	2.6 (1.0-7.2) P = 0.058	**3.9 (1.6-9.3)** P = 0.002	**3.2 (1.3-7.7)** P = 0.010	**3.8 (1.5-9.6)** P = 0.004
Upwardly mobile	1.5 (0.7-2.9) P = 0.249	1.3 (0.6-2.6) P = 0.490	1.2 (0.6-2.5) P = 0.570	1.0 (0.5-2.0) P = 0.953	0.9 (0.4-1.7) P = 0.714	1.0 (0.5-2.1) P = 0.941
Stable in high SES	1.0	1.0	1.0	1.0	1.0	1.0

Model 1 = social mobility measure + age + gender.

Model 2 = social mobility measure+ age + gender + family structure

Model 3 = social mobility measure +age + gender + family structure + paternal smoking +maternal smoking.

*Mobility from assigned socioeconomic status measured by material affluence scale to achieved social position measured by plans after graduation.

** Mobility from assigned socioeconomic status measured by father's education to achieved social position measured by plans after graduation.

#### Age and gender differences

Family structure and material affluence were associated with tawa use in the same direction for both genders but statistically significant only for girls. Also, the associations between plans after graduation and both forms of tobacco use were statistically significant only for girls. When analysed separately in two age categories, younger adolescents (13-15-year-olds) and older adolescents (16-18-year-olds), the association between MAS and tawa use was statistically significant only among the younger adolescents. There were more girls in the sample than boys (Table 1).

## Discussion

The main findings are that, first, socioeconomic differences, measured by both familial and individual SES exist in tobacco use among Ghanaian adolescents to the disadvantage of those in the lower socioeconomic groups. The differences follow the same pattern as those found in Western countries. Second, an adolescent's individual social position, measured by plans after graduation, is a stronger predictor of tobacco use than familial SES. Third, children expected to end up in adulthood in a lower SES than their families (downwardly mobile) or remained stable in the low SES are more likely to use tobacco than those children who are stable in the high SES. Fourth, the socioeconomic pattern was similar for smoking and tawa use, except that material affluence scale was related to tawa use only.

Our finding of higher probability of tobacco use among adolescents in lower SES groups is mostly in line with previous studies [4,9]. Some studies have reported high prevalence of smoking among adolescents whose parents had a low educational or occupational position [4,9]. Contrary to our expectation, familial SES measured by father's or mother's occupation and mother's education were not important predictors of an adolescent's smoking or tawa use in this study. On the other hand, adolescents of lower familial SES measured by material affluence scale were more likely to use tawa compared to those on higher material affluence scale. The traditional tawa is likely to be cheaper, more available and accessible compared to cigarettes. It is also relatively easier to hide and use without anybody noticing since it is smokeless, and perhaps the Ghanaian society is more tolerant to its use than smoking. These and other socio-cultural factors could explain in part why material affluence scale was related to tawa use but not smoking.

An adolescent's individual social position indicated by plans after graduation was strongly related with both forms of tobacco use in a similar pattern as in Western countries [5] but school performance was not. Higher prevalence of both smoking and tawa use was found among adolescents who did not have plans to continue schooling after graduation compared to those who planned to continue. Previous studies using indicators which capture the adolescents' individual SES have shown that adolescents of low individual SES are more likely to take up smoking and other health compromising behaviours, similar to our results [5,6,8,9,18]. For example, adolescents who discontinue school after the comprehensive school often engage in health-damaging behaviours typical of lower socioeconomic groups [5]. There are plausible explanations for the strong negative association between plans after graduation and tobacco use in our study. In Ghana, where there is high unemployment for even those with post-secondary education, having no plans to continue schooling after the Junior or Senior High School levels could therefore be a true sign of failure and hopelessness both for the present and the future. This may lead to low self-esteem, stress and depression and consequently result in tobacco use as a means of handling these frustrations [22]. This indicator is likely to reveal the hidden characteristic of an adolescent's individual social position independent of his or her familial status.

An adolescent's individual social position as indicated by school performance was not related with tobacco use. In Western countries, adolescents who have poor school performance have higher prevalence of smoking than those with good school performance [9]. Some explanations given for this association are that adolescents with poor school performance are likely to benefit less from health education than those of better school performance. Also, adolescents with poor school performance turn to smoking behaviour as a coping lifestyle in the face of the stress caused by educational demands [22]. In Ghana, and perhaps in most developing countries, although school performance is an important determinant of educational success and consequently future social position, factors such as gender, affordability as well as socio-cultural factors are equally important in determining the link between school performance, educational success and hence social position. It is not clear to what extent these factors account for the non-statistically significant relationship between school performance and tobacco use found in this study. Furthermore, school performance was self-reported as in most studies [e.g. [9]] but it is uncertain to what extent this might have affected the relationship between school performance and tobacco use reported here.

Adolescents living in a nuclear family had less likelihood of tobacco use than those in non-nuclear families, independent of parental smoking. Previous studies have highlighted the role of parents in the prevention of health compromising behaviours among adolescents [23-25]. Flisher et al. [26] found that among South African adolescents, not being raised by both parents was significantly associated with cigarette smoking among black and colored students. It was inversely associated with cigarette use among black students. It is likely that difference in parenting upbringing style between family structures or perhaps less parental control among adolescents not living with both parents account for this association.

There is paucity of study on inter-generational social mobility and tobacco use among adolescents. Previous study shows that health compromising behaviours such as smoking and alcohol use are more frequent among downwardly mobile and less frequent among upwardly mobile young people than their peers who have persisted in their SES of origin [17]. A recent study also found that among young people, risk behaviours like tobacco use were more prevalent among downwardly mobile or those stable compared to those upwardly mobile [18]. In our study, downwardly mobile adolescents and those staying in the low SES were more likely to use tobacco compared to those stable in the high SES similar to the previous findings. Furthermore, our findings of higher probability of tobacco use among those stable in the low SES highlight the effects of cumulative socioeconomic disadvantage over generations on adolescents' tobacco use. We did not find any statistically significant difference in tobacco use among upwardly mobile adolescents compared to their peers who were stable in the high SES.

### Strength and limitation

We used a representative sample of schools both in urban and remote rural areas in three regions which are representative of the entire country, the first study of its kind in Ghana. Some of the questions we used have also been used in other studies, for example, the Global Youth Tobacco Survey (GYTS) and the Global School-based student Health Survey (GSHS) which have been conducted in many African countries. Moreover, to the best of our knowledge our study is the first of its kind which has investigated the traditional smokeless tobacco (tawa) in Ghana.

Self-report is the only way to conduct large surveys but it could lead to recall bias or intentional miss-reporting which could affect the accuracy of the reports. However, this should not affect the relationships between SES and tobacco use among adolescents. Similar methods have been used in most previous studies [4,5]. The study was cross-sectional therefore the cause and effect relationship cannot be emphasised as an etiological conclusion, nonetheless it can be argued that at the adolescent age socioeconomic status is likely to precede tobacco use and not the reverse. During data collection an investigator was present in the classroom to address the concern of the pupils when necessary. Although we do not perceive that this might have affected the adolescents' responses, if it did, it would be more likely to have resulted in the under estimation of both the tobacco use prevalence and the socioeconomic status rather than over estimation. Our sample of students for the study was drawn from a sample of schools. The clustering of students may slightly change the standard error of our estimates, although unlikely to change neither the overall results nor the conclusion reached in this study. Due to scarce resources, only adolescents in schools have been included in this study. On the other hand, a similar pattern of responses for most of the key indicators in this study was found among a convenient sample of non-students in the same age group. Moreover, the school enrolment rate in Ghana for the age group of our respondents is high, 78.8% for Junior High Schools (Ministry of Education, Science and Sport, Ghana, 2008).

## Conclusions

Our finding of higher likelihood of tobacco use among adolescents in lower socioeconomic groups suggests that in the future there will be differences in tobacco use as well as tobacco related morbidity and mortality in Ghana between adult socioeconomic groups which will follow into health differences similar to those seen in Western countries. Furthermore, this study shows that, during adolescence, tobacco use is more influenced by individual social position than familial SES. As an adolescent's familial SES is an assigned status, its impact may be less on their health behaviours during the period of transition when adolescents move from dependent to independence. On the other hand, individual social position captures the transitional nature of adolescence as well as the social position within their peers. This study adds to the knowledge of socioeconomic differences in tobacco use among adolescents in developing countries, particularly in Africa. Health promotion and tobacco control strategies aimed at reducing adolescence tobacco use should pay attention to those of lower social and material statuses, and those in danger of discontinuing education after the basic level.

## Competing interests

The authors declare that they have no competing interests.

## Authors' contributions

DD, LK and AR were involved in the conception and design of the study. DD, SR and AR were involved in the drafting and the revising of the questionnaire. DD was the principal investigator during the data collection. DD analysed the data and drafted the manuscript. All authors were involved in the interpretation of data and the critical revision of the manuscript for important intellectual content. All authors gave final approval of the version to be published.

## Pre-publication history

The pre-publication history for this paper can be accessed here:

http://www.biomedcentral.com/1471-2458/10/758/prepub

## References

[B1] Commission on Social Determinants of Health Closing the gap in a generation: health equity through action on the social determinants of health. Final Report of the Commission on Social Determinants of Health World Health Organization, Geneva 2008 25 108 http://whqlibdoc.who.int/publications/2008/9789241563703_eng.pdf (accessed 6 March 2010)

[B2] Jarvis MJ Wardle J Marmot M, Wilkinson RG Social patterning of health behaviours: the case of cigarette smoking Social determinants of health 2006 2 Oxford: Oxford University Press 224 37

[B3] Barbeau EM Leavy-Sperounis A Balbach eds Smoking, social class, and gender: what can public health learn from the tobacco industry about disparities in smoking? Tob Control 2004 13 115 20 10.1136/tc.2003.006098 15175523 PMC1747877

[B4] Hanson MD Chen E Socioeconomic status and health behaviors in adolescence: A review of the literature J Behav Med 2007 30 3 263 285 10.1007/s10865-007-9098-3 17514418

[B5] Koivusilta LK Rimpela AH Kautiainen SM Health inequality in adolescence. Does stratification occur by familial social background, family affluence, or personal social position? BMC Public Health 2006 6 110 10.1186/1471-2458-6-110 16643660 PMC1479325

[B6] Paavola M Vartiainen E Haukkala A Smoking from adolescence to adulthood. The effects of parental and own socioeconomic status Eur J Public Health 2004 14 417 21 10.1093/eurpub/14.4.417 15542880

[B7] Koivusilta L Rimpelä A Vikat A Health behaviours and health in adolescence as predictors of educational level in adulthood: a follow-up study from Finland Soc Sci Med 2003 57 577 593 10.1016/S0277-9536(02)00405-7 12821008

[B8] Richter M Leppin A Trends in socioeconomic differences in tobacco smoking among German school children, 1994-2002 Eur J Public Health 2007 17 6 565 571 10.1093/eurpub/ckm010 17353201

[B9] Doku D Koivusilta L Rainio S Rimpelä A Socioeconomic differences in smoking among Finnish adolescents from 1977-2007 J Adolesc Health 2010 47 5 479 497 10.1016/j.jadohealth.2010.03.012 20970083

[B10] Townsend L Flisher AJ Gilreath T King G A systematic literature review of tobacco use among adults 15 years and older in sub-Saharan Africa Drug Alcohol Depend 2006 84 1 14 27 10.1016/j.drugalcdep.2005.12.008 16442750

[B11] Adu-Mireku S Prevalence of alcohol, cigarette, and marijuana use among Ghanaian senior secondary students in an urban setting J Ethn Subst Abuse 2003 2 1 53 65 10.1300/J233v02n01_05

[B12] Owusu-Dabo E Lewis S McNeill A Smoking uptake and prevalence in Ghana Tob Control 2009 18 365 370 10.1136/tc.2009.030635 19581276 PMC2745559

[B13] Owusu-Dabo E Lewis S McNeill A Smoking in Ghana: a review of tobacco industry activity Tob Control 2009 18 206 11 10.1136/tc.2009.030601 19359263 PMC2679188

[B14] Addo J Smeeth L Leon DA Smoking Patterns in Ghanaian Civil Servants: Changes Over Three Decades Int J Eviron Res Public Health 2009 6 200 208 10.3390/ijerph6010200 PMC2672323 19440277

[B15] Koivusilta L Rimpelä A Rimpelä M Health-related lifestyle in adolescence predicts adult educational level-a longitudinal study from Finland J Epidemiol Community Health 1998 52 794 801 10.1136/jech.52.12.794 10396520 PMC1756653

[B16] Power C Manor O Fox J Health and Class: The Early Years 1990 Chapman and Hall, London

[B17] Karvonen S Rimpelä AH Rimpelä MK Social mobility and health related behaviours in young people J Epidemiol Community Health 1999 53 4 211 217 10.1136/jech.53.4.211 10396546 PMC1756866

[B18] Hart C McConnachie A Upton M Watt G Risk factors in the Midspan family study by social class in childhood and adulthood Int J Epidemiol 2008 1 11 10.1093/ije/dyn052 18356195

[B19] Doku D Koivusilta L Rimpelä A Indicators for measuring material affluence of adolescents in health inequality research in developing countries Child Ind Res 2010 3 243 260 Published online first:3 October 2009 10.1007/s12187-009-9045-7 PMC2837228 20339572

[B20] Townsend P Deprivation J Soc Policy 1987 16 125 146 10.1017/S0047279400020341

[B21] Currie CE Elton RA Todd J Platt S Indicators of socioeconomic status for adolescents: the WHO Health Behaviour in School-aged Children Survey Health Educ Res 1997 12 385 397 10.1093/her/12.3.385 10174221

[B22] Koval JJ Pedersson LL Stress-coping and other psychosocial risk factors: A model for smoking in grade 6 students Addict Behav 1999 24 207 218 10.1016/S0306-4603(98)00037-9 10336102

[B23] McMunn AM Nazroo JY Marmot MG Children's emotional and behavioural well-being and the family environment: findings from the Health Survey for England Soc Sci Med 2001 53 423 440 10.1016/S0277-9536(00)00346-4 11459394

[B24] Harland P Reijneveld SA Brugman E Family factors and life events as risk factors for behavioural and emotional problems in children Eur Child Adolesc Psychiat 2002 11 176 84 10.1007/s00787-002-0277-z 12444427

[B25] Rainio US Rimpelä AH Home-based sourcing of tobacco among adolescents Prev Med 2009 48 378 382 10.1016/j.ypmed.2009.01.016 19463486

[B26] Flisher AJ Parry CDH Evans J Muller M Lombard C Substance use by adolescents in Cape Town: Prevalence and correlates J Adolesc Health 2003 32 58 65 10.1016/S1054-139X(02)00445-7 12507802

